# Efficacy and safety of narrowband ultraviolet B phototherapy for prurigo nodularis: a tertiary center experience^☆^

**DOI:** 10.1016/j.abd.2024.02.007

**Published:** 2024-10-31

**Authors:** Esra Agaoglu, Hilal Kaya Erdogan, Ersoy Acer, Zeynep Nurhan Saracoglu

**Affiliations:** Department of Dermatology, Faculty of Medicine, Eskisehir Osmangazi University, Eskisehir, Turkey

**Keywords:** Phototherapy, Prurigo, Pruritus, Ultraviolet therapy

## Abstract

**Background:**

Prurigo nodularis is a chronic pruritic dermatosis and narrowband-UVB (NB-UVB) phototherapy is considered an effective and safe treatment option in patients with multiple comorbidities.

**Objective:**

In this study, the authors aimed to evaluate the efficacy and safety of NB-UVB phototherapy in the management of prurigo nodularis and to compare response rates according to lesions localization.

**Methods:**

Thirty prurigo nodularis patients who had been treated with NB-UVB phototherapy were included in this study. The data for this study were retrieved retrospectively from patient follow-up forms in the phototherapy unit.

**Results:**

NB-UVB phototherapy led to a complete response (CR) in 24 (80%) patients while partial response (PR) was achieved in 6 (20%) patients. Regarding prurigo nodularis localization, the CR rate was statistically higher in those with diffuse and central involvement (p < 0.05). Erythema and/or pruritus were observed in 4 (13.3%) patients with prurigo nodularis.

**Study limitations:**

This study is limited because it is single-centered and has a retrospective design.

**Conclusions:**

NB-UVB phototherapy is an effective and safe treatment option for prurigo nodularis patients especially those with multiple comorbidities and using several medications. Patients with diffuse and central involvement may respond better to phototherapy than those with peripheral involvement.

## Introduction

Prurigo nodularis is a chronic inflammatory dermatosis characterized by intensely pruritic hyperkeratotic nodular lesions. These nodules tend to be symmetrically distributed on the extensor surfaces of the extremities or trunk, and rarely affect the palms and face. Intense pruritus experienced by patients results in crusting, bleeding, or pain in nodular lesions. Although the exact cause of prurigo nodularis is unknown, it is thought that neuronal sensitization resulting from neuroimmune dysregulation causes a repetitive itch-scratch cycle. Chronic pruritus in prurigo nodularis decreases the quality of life and can be associated with many comorbid conditions including psychiatric, endocrine, cardiovascular, and renal disorders as well as malignancies.^1^, ^2^

The management of prurigo nodularis is often challenging. Patients' comorbid conditions and side effects of therapies should be considered for individualized treatment. Topical antiprurutic agents such as corticosteroids, calcineurin inhibitors and anesthetic therapies, intralesional corticosteroids or systemic antipruritic therapies including Ultraviolet (UV) phototherapy, gabapentinoids, antidepressants, opioid antagonists and immunosuppressants are recommended treatment options for prurigo nodularis.^1^, ^3^

UV therapy has been shown to reduce pruritus in many dermatological diseases by interrupting the itch-scratch cycle with its anti-inflammatory and neuromodulatory effects.^4^ Among present phototherapy options, Narrowband (NB) UVB (NB-UVB) phototherapy emitting wavelength between 310 and 315 nm has been suggested as a well-tolerated and first-line treatment for patients with prurigo nodularis who are unresponsive to topical treatments.^3^, ^5^, ^6^, ^7^

The aim of this study is to evaluate the effectiveness and safety of NB-UVB phototherapy for the management of prurigo nodularis according to the localization.

## Materials-methods

### Patients

This study included 30 prurigo nodularis patients treated with NB-UVB who were followed up in the phototherapy unit between 2011 and 2021. The study protocol was approved by the Institutional Ethics Committee. The phototherapy data were evaluated retrospectively from the follow-up forms of the patients. Patients who had a clinical and/or biopsy-proven diagnosis of prurigo nodularis were included.

Before phototherapy, patients were evaluated for cutaneous malignancies and photosensitive drug use. For each patient, demographic and clinical data including age, gender, comorbidities, previous treatments, duration of disease, localization of lesions, and skin type according to Fitzpatrick's classification were documented. The required wash-out period before phototherapy was 2‒4 weeks for topical and/or systemic treatments. Comorbidities of the patients were classified as psychiatric diseases, endocrine-metabolic diseases (diabetes mellitus, chronic kidney disease, thyroid-parathyroid diseases), cardiovascular diseases (hypertension, coronary artery disease, congestive heart disease) and malignancies. Phototherapy-related variables, the number of sessions, follow-up period in phototherapy, first-maximum dose of phototherapy, cumulative dose, response to phototherapy and relapse rates were included.

### Distribution of lesions

Localization of lesions was classified as central, peripheral, or diffuse before phototherapy. The lesions limited to extremities were classified as peripheral, involvement of the trunk was classified as central, and involvement of both trunk and extremities was classified as diffuse involvement. The mean number of sessions and the mean cumulative doses were also recorded according to the localization.

### Phototherapy protocol

NB-UVB treatment was applied with the Daavlin Spectra 305/350 model UV device to the patients included in this study. NB-UVB phototherapy was initially started with 70% of the minimal erythema dose and treatment continued with 10%‒20% dose increases at each subsequent session. While the phototherapy dose continued with a 10% increase in patients who developed minimal erythema, the dose was not increased in patients who developed moderate erythema. Phototherapy was discontinued in patients who developed severe erythema and/or edema. After severe erythema subsided the phototherapy dose started with 50% of the last dose and continued with 10% increments. NB-UVB therapy was administered three times per week and was continued twice or once per week when patients had a clinical response at monthly visits. Emollient creams were used in all of the patients from the first session of phototherapy. The patients were followed for 12 months after cessation of their treatment regarding the development of any recurrence.

### Evaluation of treatment efficacy

Response to treatment was evaluated in patients who received NB-UVB phototherapy for at least 3 months. At monthly clinic visits after starting phototherapy, the patients were evaluated subjectively whether the intensity of itching had decreased, and the regression of lesions was assessed by the clinician. According to the distribution of lesions; patients with more than 90% clearance were considered as complete response (CR) and those with between 50% and 90% were considered as partial response (PR).^8^ The age of the patients (<65 years vs. ≥65 years), duration of disease (<2 years vs. ≥2 years), distribution of lesions, previous treatments, comorbidities, mean number of sessions, cumulative doses and relapse rates were compared in both patients who had CR and PR. Relapse was defined as an increase in the number of nodules after it had improved or cleared completely in a 1 year follow-up period.

### Statistical analysis

Descriptive statistics for continuous variables were shown as mean ± standard deviation or median (minimum‒maximum), and categorical variables were shown as the number of cases (%). Normality analysis of numerical data was calculated through Kolmogorov Smirnov test. It was determined that the data did not show a normal distribution. The median (minimum‒maximum) values of the data were given with Chi-Square and Mann–Whitney *U* tests were used for statistical evaluations; p < 0.05 was accepted as statistical significance. The data analyses were performed with SPSS Statistics Pack version 25.0 (IBM, Armonk, NY).

## Results

### Demographics, clinical and treatment features of prurigo nodularis

A total of 30 patients treated with NB-UVB with the diagnosis of prurigo nodularis were included in this study. Twenty-one (70%) of the patients were female and 9 (30%) of the patients were male. The mean age of the patients was 55.53 ± 14.88 (range: 18‒76) years. Nine (30%) of the patients were 65 years of age and older. The mean duration of the disease was 27.30 ± 20.26 months. The diagnosis of prurigo nodularis was confirmed with histopathological examination in 14 (46.6%) of the patients. In evaluation of the localization of lesions: peripheral involvement was detected in 15 (50.0%) of the patients, diffuse involvement in 11 (36.6%) patients, and central involvement in 4 (13.3%) patients. Prior to phototherapy, topical steroids and antihistamines were administered in 21 (70.0%) of the patients, topical steroids in 5 (16.6%) of the patients and both topical and systemic steroids in 4 (13.3%) of the patients. Eight (26.6%) of the patients had either an atopic diathesis or atopic predisposition. Two (6.6%) of the patients had concomitant psoriasis vulgaris. No accompanying infectious disease was detected. An accompanying disease was documented in 24 cases (80%). Type 2 diabetes mellitus and psychiatric disorders were the most common comorbidities detected in 14 (46.6%) and 12 (40.0%) of the patients respectively. Of the patients with psychiatric diseases, 9 (75%) were diagnosed with anxiety disorder and 3 (25%) with depressive disorder (Table 1).

**Table 1:** Demographic and clinical features of patients with prurigo nodularis.

**Gender**	
Females (%)	21 (70%)
Males (%)	9 (30%)
**Age**	
Mean ± SD	55.53 ± 14.88
Range	18‒76
**Mean duration of disease ± SD (months)**	27.30 ± 20.26
**Histopathological confirmation of the diagnosis**	14 (46.6%)
**Involvement**	
Peripheral	15 (50.0%)
Diffuse	11 (36.6%)
Central	4 (13.3%)
**Skin types according to Fitzpatrick’s classification**	Ⅰ: 1, Ⅱ:15, Ⅲ:14
**Previous treatment, n (%)**	
Topical steroid	5 (16.6%)
Topical steroid + antihistamines	21 (70.0%)
Topical + systemic steroid	4 (13.3%)
**Comorbidities**	23 (76.6%)
Psychiatric disorders	12 (40.0%)
Metabolic-endocrine disorders	19 (63.3%)
Diabetes mellitus	14 (46.6%)
Chronic kidney disease	1 (3.3%)
Thyroid-parathyroid disease	4 (13.3%)
Cardiovascular disease	4 (13.3%)
Malignancy (breast cancer: 2, lung cancer: 1)	3 (10.0%)

SD, Standard Deviation.

### Phototherapeutic data of the patients

The follow-up period in phototherapy was 5.6 ± 3.3 months (range: 3‒16). The mean number of sessions was 45.7 ± 22.8 (range: 14‒94), with a mean cumulative dose of 2001 ± 451 J/cm^2^ (range: 8.7‒250.1 J/cm^2^). CR was achieved in 24 (80%) of the patients while PR was achieved in 6 (20%) of the patients. Erythema and/or pruritus were observed in 4 (13.3%) of the patients as the most common side effect during the phototherapy. None of the patients discontinued the treatment due to side effects. Topical corticosteroids and emollients were used for erythema and/or pruritus. Follow-up data were available for 21 patients and relapse was observed in 5 (23.8%) of them during the 1-year follow-up (Table 2).

**Table 2:** Phototherapeutic data of the patients with prurigo nodularis.

		**NB-UVB**	
		**Mean ± SD**	**Median (range)**
**Number of sessions**		45.7 ± 22.8	41 (14‒94)
**Follow-up period in phototherapy (months)**		5.6 ± 3.3	5 (3‒16)
**First dose (J/cm^2^)**		289.6 ± 56.5	300 (200‒450)
**Maximum dose (J/cm^2^)**		2001 ± 451	2000 (1080‒3300)
**Cumulative dose (J/cm^2^)**		69.2 ± 57.1	58.7 (8.7‒250.1)
		n (%)	
**Treatment response**	Complete response (CR)	24 (80%)	
	Partial response (PR)	6 (20%)	
**Side effects**		4 (13.3%)	
**Relapse**		5 (23.8%)	

SD, Standard Deviation.

In the evaluation of the response to phototherapy according to sociodemographic and clinical features; no statistically significant differences between CR and PR rates were found in terms of geriatric patients, gender, duration of disease, previous treatments, comorbidities, mean number of sessions, mean cumulative dose and side effects (Table 3).

**Table 3:** Clinical and treatment features of patients with prurigo nodularis according to phototherapy response.

	**Complete response (CR)**	**Partial response (PR)**	**p**
**Age**			0.842
<65 years	17 (81.0%)	4 (19.0%)	
≥65 years	7 (77.8%)	2 (22.2%)	
**Gender**			0.426
Men	8 (88.9%)	1 (11.1%)	
Women	16 (76.2%)	5 (23.8%)	
**Duration of disease**			0.272
<2 years	10 (71.4%)	4 (28.6%)	
≥2 years	14 (87.5%)	2 (12.5%)	
**Previous treatments**			0.551
Topical steroid	4 (80.0%)	1 (20.0%)	
Topical steroid + antihistamines	16 (76.2%)	5 (23.8%)	
Topical steroid + systemic steroid	4 (100.0%)	0 (0.0%)	
**Comorbidities**			0.709
**Psychological disorders**	10 (83.3%)	2 (16.7%)	
**Metabolic-endocrine disorders**			
Diabetes mellitus	10 (71.4%)	4 (28.6%)	0.522
Thyroid- parathyroid disease	3 (75.0%)	1 (25.0%)	0.788
**Cardiovascular disease**	4 (80.0%)	1 (20.0%)	0.702
**Malignancy**	2 (66.7%)	1 (33.3%)	0.501
**Mean number of sessions (±SD)**	48.21 ± 23.58	35.83 ± 17.65	0.299
**Mean cumulative dose (±SD) (J/cm^2^)**	74.57 ± 60.73	47.95 ± 36.06	0.325
**Side effects**	4 (100.0%)	0 (0.0%)	0.283

SD, Standard Deviation.

Patients with diffuse and central involvement had statistically higher CR rates than patients with peripheral involvement (p < 0.05). Mean number of sessions and mean cumulative dose of patients with diffuse and central involvement were higher than those of patients with peripheral involvement, however; this difference was not statistically significant (Table 4).

**Table 4:** Phototherapeutic data of the patients according to the localization of the lesions.

	**Peripheral**	**Diffuse**	**Central**	**p**
**Clinical response**	CR: 9 (60.0%)	CR: 11 (100.0%)	CR: 4 (100.0%)	**0.024**
	PR: 6 (40.0%)	PR: -	PR: -	
**Mean number of sessions (±SD)**	37.20 ± 19.29	52.45 ± 24.50	59.25 ± 22.72	0.064
**Mean cumulative dose (±SD) (J/cm^2^)**	49.29 ± 37.30	88.62 ± 77.23	90.82 ± 34.83	0.057

SD, Standard Deviation.

## Discussion

Prurigo nodularis is a chronic disease characterized by the presence of multiple symmetrically distributed hyperkeratotic itchy nodules mainly on the extremities and trunk.^2^ A cutaneous reaction pattern caused by a vicious cycle of chronic pruritus followed by recurrent scratching is involved in the pathogenesis of prurigo nodularis.^3^, ^9^, ^10^ Usually it affects middle-aged adults in the fifth and sixth decades of life and seems to show a slight female predominance.^2^ In the present study, the epidemiological characteristics of the patients in terms of age and gender were similar to the studies reported in the literature.^11^, ^12^

While chronic dermatoses cause a decrease in quality of life and predisposition to psychiatric comorbidities, they also play a role in the etiopathogenesis of these diseases. It is demonstrated that significant psychiatric morbidity such as anxiety and depressive disorders are common in patients with prurigo nodularis.^2^, ^13^, ^14^ A recent study revealed that 44% of patients with prurigo nodularis had at least one psychiatric comorbidity, particularly mood and anxiety disorders.^13^ Although some antidepressants are recommended due to potential antipruritic effects in the treatment of prurigo nodularis,^3^ in a study by Jørgensen et al., they reported that usage of anxiolytics and antidepressants was significantly higher among patients with prurigo nodularis compared to age and gender-matched controls.^15^ Additionally, Arrieta et al. reported that the most commonly associated comorbidities were psychological disorders which were detected in 72% of the patients with prurigo nodularis.^5^ In the present study, 12 (40.0%) of the patients with prurigo nodularis had concomitant psychiatric comorbidity and the most common disorder was anxiety disorder.

Although prurigo nodularis is most commonly associated with inflammatory dermatoses such as atopic dermatitis, several metabolic and endocrine disorders may also accompany the disease.^2^ Patients with prurigo nodularis has an increased risk of diabetes, chronic kidney disease and cardiovascular conditions.^11^ It is postulated that endocrine and metabolic dysfunction affects over half of patients with prurigo nodularis.^16^, ^17^ Severe pruritus and impaired wound healing due to underlying metabolic disorders may play a role in the development of prurigo nodularis.^2^, ^18^ A study by Winhoven et al. reported that diabetes mellitus and thyroid diseases were the most commonly associated metabolic comorbidities in patients with prurigo nodularis.^17^ Similarly, type 2 diabetes mellitus and thyroid diseases were the most common metabolic-endocrine comorbidities observed in the patients. Boozalis et al. documented that prurigo nodularis was associated with cardiovascular conditions such as hypertension, ischemic heart disease and congestive heart failure and this was statistically significant. They suggested that chronic pruritus is common in diabetes mellitus and chronic renal failure therefore, observation of pruritus may also indirectly increase in hypertension which frequently accompanies these diseases.^11^

UV phototherapy has long been used as a well-tolerated skin-directed therapy for the management of many pruritic inflammatory dermatologic conditions including psoriasis, atopic dermatitis, lichen planus, pityriasis lichenoides and cutaneous T-cell lymphoma.^19^ Although it is well-documented that phototherapy is effective at reducing pruritus in these conditions, the mechanism of action is not completely understood.^20^, ^21^ It is suggested that phototherapy decreases pruritus by suppressing the immune system, reduces the inflammation of the skin and also directly affects neural signaling.^20^ Generally, several UV phototherapy modalities consisting of Ultraviolet A (UVA), bath psoralen plus UVA (PUVA), broad-band Ultraviolet B (BB-UVB) and NB-UVB are the preferred options for prurigo nodularis.^10^ It is well documented that T-lymphocytes, especially Interleukin (IL)-31, mast cells and eosinophils play a role in the immune dysregulation of disease.^3^ IL-31 is a T-lymphocyte-derived cytokine that directly stimulates sensory neurons for the generation of pruritus.^22^ In a study that compared the skin biopsies from patients with prurigo nodularis and healthy skin, it is demonstrated that high IL-31 levels were detected in the skin of prurigo patients.^23^ Neural sensitization to itch and neurogenic inflammation also contribute to pathogenesis of prurigo nodularis. Nerve Growth Factor (NGF) and Calcitonin Gene-Related Peptide (CGRP) are the main neuropeptides that are upregulated in pruritic skin. Phototherapy is thought to act by reducing the number of epidermal and dermal nerve fibers and decreasing neuropeptide expression in the skin.^19^, ^20^, ^24^ It is demonstrated that UVB radiation reduces NGF, dermal CGRP levels, and also IL-31 levels with its immunosuppressive effect.^25^, ^26^ Additionally, the antipruritic effect of UVB treatment is attributed to inhibiting the release of granules from mast cells.^8^

Several studies documented variable results of clinical response in NB-UVB phototherapy in the treatment of prurigo nodularis alone or in combination with systemic treatments. However, publications on NB-UVB phototherapy in prurigo nodularis consist mainly of case series or nonrandomized trials with small sample sizes. Additionally, the phototherapy protocol and evaluation of response to phototherapy differ between studies in the literature.^5^, ^6^, ^7^, ^27^, ^28^ In the present study, NB-UVB treatment induce CR in 24 (80%) of 30 prurigo nodularis patients with a mean cumulative dose of 74.5 J/cm^2^ after a mean number of 48.2 sessions. PR was achieved in 6 (20%) of 30 patients with a mean cumulative dose of 47.9 J/cm^2^ after a mean number of 35.8 sessions. Fernandiz et al. reported 4 patients with prurigo nodularis who were treated with sequential combined therapy with NB-UVB and thalidomide, all achieved a complete remission with a mean cumulative dose of 40.5 J/cm^2^ after 32 sessions with NB-UVB phototherapy.^7^ A study by Carrascosa et al., 15 patients with prurigo nodularis were treated with 22 therapeutic cycles of NB-UVB phototherapy, and > 80% regression was obtained in 5 (67%) of the patients with a mean cumulative dose of 26.2 J/cm^2^.^27^ Tamagawa-Mineoka et al. evaluated the treatment response of NB-UVB phototherapy once weekly in 10 patients with prurigo nodularis. The mean duration of prurigo nodularis was 5.6 years and all patients had a notable improvement with a mean cumulative dose of 23.8 J/cm^2^ after 24.3 sessions.^6^ Arrieta et al. used NB-UVB for 34 patients with prurigo nodularis who had a history of 5 years of disease. In that study after 3 months of phototherapy, >75% regression of the lesions was observed in 54.5% of the patients.^5^ In the present study, a higher number of sessions and cumulative doses were given compared to the literature, but there were no statistically significant differences between CR and PR rates. Although the duration of the disease in patients was shorter compared to studies in the literature,^5^, ^6^ >90% clinical response was achieved in 80% of the patients with higher sessions and cumulative doses of NB-UVB phototherapy.

There is no study comparing response rates of phototherapy according to the localization of prurigo nodularis lesions. It is well known that phototherapy has been effective at controlling pruritus in patients with generalized lesions as well as patients with multiple comorbid conditions.^20^, ^29^, ^30^ Generalized prurigo nodularis is extremely difficult to treat and applying the topical treatments to the widespread areas of the body may be challenging. In generalized cases, NB-UVB phototherapy encourages the patients' compliance with the therapy with its low side effect profile.^31^ In this study, patients with diffuse and central involvement had higher CR rates than peripheral involvement. Although it was not statistically significant, a higher mean number of sessions and higher cumulative doses were obtained in patients with diffuse and central involvement. Therefore, increasing both the number of sessions and cumulative doses of NB-UVB phototherapy, which has a low risk of side effects, may play a role in achieving higher CR rates in patients with diffuse or central involvement compared to acral involvement.

In the present study, the most common side effects during phototherapy were erythema and/or pruritus, with a frequency similar to the studies in the literature,^5^, ^6^ and no side effects severe enough to discontinue the treatment were observed.

Limitations of this study include that it was a single-center retrospective design. In this study, comorbidities or related conditions accompanying prurigo nodularis reflect only the Turkish population. Additionally, among the phototherapy options, only NB-UVB therapy was evaluated in the treatment of prurigo nodularis.

## Conclusion

Prurigo nodularis is associated with several comorbidities such as diabetes mellitus and psychiatric disorders. NB-UVB phototherapy treatment is an effective and safe option when there is no satisfactory response to topical treatment in prurigo nodularis patients with comorbidities and multiple medications. Patients with diffuse and central involvement may achieve higher CR rates than patients with peripheral involvement. Wider scaled and prospective studies are needed to investigate the efficacy and safety of NB-UVB phototherapy in the treatment of prurigo nodularis.

## Financial support

None declared.

## Authors’ contributions

Esra Agaoglu: Approval of the final version of the manuscript; Design and planning of the study; Drafting and editing of the manuscript; Collection, analysis, and interpretation of data; Effective participation in research orientation; Intellectual participation in the propaedeutic and/or therapeutic conduct of the studied cases; Critical review of the literature; Critical review of the manuscript.

Hilal Kaya Erdogan: Approval of the final version of the manuscript; Critical literature review; Data collection, analysis, and interpretation; Effective participation in research orientation; Intellectual participation in propaedeutic and/or therapeutic management of studied cases; Manuscript critical review; Preparation and writing of the manuscript; Study conception and planning.

Ersoy Acer: Approval of the final version of the manuscript; Critical literature review; Data collection, analysis, and interpretation; Effective participation in research orientation; Intellectual participation in propaedeutic and/or therapeutic management of studied cases; Manuscript critical review.

Zeynep Nurhan Saracoglu: Approval of the final version of the manuscript; Critical literature review; Data collection, analysis, and interpretation; Effective participation in research orientation; Intellectual participation in propaedeutic and/or therapeutic management of studied cases; Manuscript critical review.

## Conflicts of interest

None declared.
